# Concerted and Independent Evolution of Control Regions 1 and 2 of Water Monitor Lizards (*Varanus salvator macromaculatus*) and Different Phylogenetic Informative Markers

**DOI:** 10.3390/ani12020148

**Published:** 2022-01-08

**Authors:** Watcharaporn Thapana, Nattakan Ariyaraphong, Parinya Wongtienchai, Nararat Laopichienpong, Worapong Singchat, Thitipong Panthum, Syed Farhan Ahmad, Ekaphan Kraichak, Narongrit Muangmai, Prateep Duengkae, Kornsorn Srikulnath

**Affiliations:** 1 Animal Genomics and Bioresource Research Center (AGB Research Center), Faculty of Science, Kasetsart University, 50 Ngamwongwan, Bangkok 10900, Thailand; golf_foureye@hotmail.com (W.T.); nattakan.ari@ku.th (N.A.); nararat.l@ku.th (N.L.); fsciwos@ku.ac.th (W.S.); thitipong.pa@ku.th (T.P.); syedfarhan.a@ku.th (S.F.A.); fforptd@ku.ac.th (P.D.); 2 Laboratory of Animal Cytogenetics and Comparative Genomics (ACCG), Department of Genetics, Faculty of Science, Kasetsart University, 50 Ngamwongwan, Bangkok 10900, Thailand; parinya_wongtienchai@hotmail.com; 3 Special Research Unit for Wildlife Genomics (SRUWG), Department of Forest Biology, Faculty of Forestry, Kasetsart University, 50 Ngamwongwan, Bangkok 10900, Thailand; 4 Department of Botany, Faculty of Science, Kasetsart University, 50 Ngamwongwan, Bangkok 10900, Thailand; ekaphan.k@ku.th; 5 Department of Fishery Biology, Faculty of Fisheries, Kasetsart University, 50 Ngamwongwan, Bangkok 10900, Thailand; ffisnrm@ku.ac.th; 6 Amphibian Research Center, Hiroshima University, 1-3-1, Kagamiyama, Higashihiroshima 739-8526, Japan

**Keywords:** varanid, control region, ortholog, paralog

## Abstract

**Simple Summary:**

The evolutionary patterns and phylogenetic utility of duplicate control regions (CRs) in 72 individuals of *Varanus salvator macromaculatus* and other varanids have been observed. Divergence of the two CRs from each individual revealed a pattern of independent evolution in CRs of varanid lineage. This study is a first step towards developing new phylogenetic evolutionary models of the varanid lineage, with accurate evolutionary inferences to provide basic insights into the biology of mitogenomes.

**Abstract:**

Duplicate control regions (CRs) have been observed in the mitochondrial genomes (mitogenomes) of most varanids. Duplicate CRs have evolved in either concerted or independent evolution in vertebrates, but whether an evolutionary pattern exists in varanids remains unknown. Therefore, we conducted this study to analyze the evolutionary patterns and phylogenetic utilities of duplicate CRs in 72 individuals of *Varanus salvator macromaculatus* and other varanids. Sequence analyses and phylogenetic relationships revealed that divergence between orthologous copies from different individuals was lower than in paralogous copies from the same individual, suggesting an independent evolution of the two CRs. Distinct trees and recombination testing derived from CR1 and CR2 suggested that recombination events occurred between CRs during the evolutionary process. A comparison of substitution saturation showed the potential of CR2 as a phylogenetic marker. By contrast, duplicate CRs of the four examined varanids had similar sequences within species, suggesting typical characteristics of concerted evolution. The results provide a better understanding of the molecular evolutionary processes related to the mitogenomes of the varanid lineage.

## 1. Introduction

The mitochondrial control region (mtCR) is a major noncoding segment of the vertebrate mitochondrial genome (mitogenome). The region includes the displacement loop (D-loop), which comprises the third strand of DNA, thus creating a semi-stable structure [1]. The mtCR plays an important role in transcriptional and translational regulation of protein-coding sequences, or it serves as the origin of DNA replication [2]. The nucleotide CR sequence is the most rapidly evolving region of the mitogenome, and it lacks coding sequences; thus, it is widely used as a molecular marker in population genetics, phylogenetic studies, and phylogeographic studies [3,4,5]. Vertebrates such as birds, snakes, turtles, and fish exhibit segmental duplications within the CR or an entire duplication of the CR, leading to the formation of repeats or possible homogenization between the duplicated copies of CR [6,7,8].

GenBank contains 17,489 complete mitogenomes for squamate reptiles (as of April 2021, http://www.ncbi.nlm.nih.gov/genome), with several duplicate CRs observed in varanids and snakes [6,7]. A comparison between two CRs (CR1 and CR2) revealed identical or highly similar nucleotide sequences, similar to the concerted evolution as found in Bothidae and Samaridae [9]. By contrast, orthologous copies of duplicate CRs from different species such as in varanid, gecko lizard, and platysternid lineages, are genetically closer to each other than to paralogous copies of duplicate CRs (CR1 and CR2) within the same species [6]. This might be a result of the independent evolution of the two copies after an ancient duplication event, although the mechanism behind such an event is not clearly understood [8].

Varanids or monitor lizards comprise a single extant genus, *Varanus*, within the family Varanidae. To date, around 80 extant species have been described and distributed in Afro-Arabia, Western to Southeast Asia, the Indonesian Archipelago, Papua New Guinea, and Australia [10]. Mitogenomes of the Komodo dragon (*V. komodoensis*; Ouwens 1912) [11,12] and Nile monitor (*V. niloticus*; Linnaeus 1758) [13,14] have unique gene organization features. Genes between the NADH dehydrogenase subunit 6 (*ND6*) gene and proline tRNA gene are extensively shuffled, and the CR has been duplicated in an ancestral varanid lineage during the Paleocene age or earlier [15]. This is consistent with the Cenozoic over-water dispersal of Southeast Asian varanids, such as the water monitor (*V. salvator macromaculatus*; Deraniyagala 1944) [16] across the Indonesian Archipelago and Komodo dragon (*V. komodoensis*) [15]. The presence of duplicate CRs in varanid mitogenomes is an intriguing structural phenomenon and raises basic questions concerning how the nucleotide sequences of duplicate CRs remained similar over time. Variations in CRs at the population and species level in varanids have not been fully elucidated [5,17]. In light of this scenario, we propose two hypotheses: (1) orthologous copies of duplicate CRs in different individuals are genetically similar due to independent evolution, or (2) two CRs (CR1 and CR2) as paralogous copies exhibit identical or highly similar nucleotide sequences from concerted evolution. To characterize the variations in varanid CRs, we conducted this study to analyze the CR sequences of four varanids, namely *V. salvator* (*V. salvator macromaculatus* and *V.* *salvator komaini*), *V. exanthematicus*, *V. komodoensis* and *V. niloticus*, and 72 water monitors (*V. salvator macromaculatus*) (Table 1). We also compared and analyzed the sequence variations of mtCRs. These analyses have important implications in the selection of priority mitochondrial regions to assess the evolution and genetic diversity of varanid populations.

## 2. Materials and Methods

### 2.1. Specimen Collection and DNA Extraction

Blood specimens of water monitors (*V. salvator macromaculatus*) were collected from the ventral tail vein using a 23-gauge needle attached to a 2 mL disposable syringe containing 10 mM ethylenediaminetetraacetic acid for DNA extraction as previously reported by Wongtienchai et al. [5] (Supplementary Table S1). Samples were collected from 47 individuals at the Bang Kachao Peninsula, Samut Prakan, (13°59′2″ N, 99°59′38″ E) and from 25 individuals at Varanus Farm Kamphaeng Saen, Nakhon Pathom (14°00′59.9″ N, 99°57′46.8″ E). Permission was granted by the Sri Nakhon Khuean Khan Park (Royal Forest Department, Ministry of Natural Resources and Environment) and Kasetsart University (0909.6/15779). All experimental procedures involving animals conformed to the guidelines established by the Animal Care Committee of Kasetsart University, Thailand. Total genomic DNA was extracted according to the standard salting-out protocol, as previously described [18]. DNA quality and concentration were determined using 1% agarose gel electrophoresis and spectrophotometry.

### 2.2. CR1 Sequencing

The positions of duplicate CRs were determined from the locations of tRNA genes (tRNAPro and tRNAVal for CR1; tRNATyr and tRNASer for CR2) as the flanking regions of CR1 and CR2 in the mitogenome of *V. salvator* (GenBank accession number: EU747731). The CR1 fragments were amplified using the following primers: VSA_CR1 F (5′-ATTAATACCCAATTTTCCTTGCTC-3′) and VSA_CR1 R (5′-GCCCAGTGACCATTAATATCAACT-3′), which were designed based on five varanid mtDNA sequences, namely *V. salvator macromaculatus* (GenBank accession number: AB980995), *V. salvator komaini* (GenBank accession number: AB980996), *Varanus exanthematicus* (GenBank accession number: AB738957), *Varanus komodoensis* (GenBank accession number: AB080276), and *Varanus niloticus* (GenBank accession number: AB185327). The positions of all primers were located in tRNA genes (tRNAPro and tRNAVal for CR1; tRNATyr and tRNASer for CR2) that are highly conserved along all varanus mitogenomes; therefore, entire sequences of both CRs were collected. Polymerase chain reaction (PCR) amplification was performed using 20 μL of 1× ThermoPol buffer containing 1.5 mM MgCl_2_, 0.2 dNTPs, 5.0 μM primers, 0.5 U of *Taq* polymerase (Apsalagen Co., Ltd., Bangkok, Thailand), and 25 ng of genomic DNA. The PCR conditions were as follows: initial denaturation at 94 °C for 3 min, followed by 35 cycles at 94 °C for 30 s, 57 °C for 30 s, and 72 °C for 40 s, and a final extension at 72 °C for 5 min [5]. The PCR products were separated via electrophoresis on 1% agarose gels, and they were then cloned using the pGEM^®^-T Easy vector (Promega Corporation, Madison, WI, USA). Nucleotide sequences of DNA fragments were determined using the DNA sequencing service of First Base Laboratories Sdn Bhd (Seri Kembangan, Selangor, Malaysia). BLASTn programs (http://blast.ncbi.nlm.nih.gov/Blast.cgi) were used to search nucleotide sequences in the National Center for Biotechnology Information database to confirm the identities of amplified DNA fragments. The generated sequences were deposited in the DNA Data Bank of Japan. The mitochondrial CR2 dataset used in our previous study was retrieved from the database [5].

### 2.3. Positional Annotation

Three functional regions, including the terminal-associated sequence (TAS), central conserved domain (CD), and conserved sequence blocks (CSB), were tentatively investigated in both CRs by recognizing sequences similar to those found in other vertebrates [3,6,12]. Variable number of tandem repeats (VNTRs) have been reported to exist only at the 3′ end of both CRs [19,20]. Tandem repeat sequences, including the motif, length of repeats, and copy number in the CR region, were investigated using the Tandem Repeats Finder 4.09 program [21].

### 2.4. Comparison of Genetic Variability Based on CR Sequences at the Population Level

Multiple sequence alignment was performed for 72 sequences in both CRs using the default parameters of Molecular Evolutionary Genetics Analysis X (MEGAX) software (Center for Evolutionary Functional Genomics, The Biodesign Institute, Tempe, PA, USA; [22]). Estimates of haplotype (*h*), nucleotide (π) diversity [23], and number of haplotypes (H) were calculated based on CR1 and CR2 sequences, as implemented in DnaSP version 6 [24]. A statistical parsimony network of consensus sequences was constructed using the Templeton, Crandall, and Sing (TCS) algorithm implemented in PopART version 1.7. to address haplotype grouping [25]. The mitochondrial CR2 dataset employed in our previous study [5] was used for all analyses, similar to the CR1 dataset. The means and standard deviations of *h* and π diversity of both CRs were used to calculate t-statistics and *p*-values for two-sample *t*-test comparisons, following the formula in [26] in R version 4.0.3 [27].

### 2.5. Phylogenetic Analysis Based on Mitochondrial CR1 and CR2 Sequences at the Species Level

Substitution saturation decreases the amount of phylogenetic signal to the point that sequence similarities could be a result of chance alone rather than homology. Consequently, when saturation is achieved, the phylogenetic signal is lost, and the sequences no longer reveal the underlying evolutionary mechanisms [28]. The saturation of substitutions was evaluated by plotting the number of transitions (s) and transversions (v) against the K80 [29] sequence divergences as well as by comparing the information entropy-based index (I_ss_) with critical values (I_ss.c_) [30,31], as implemented in DAMBE7 [32]. If I_ss_ is significantly lower than I_ss.c_, the sequences do not experience substitution saturation. Phylogenetic analyses were performed using CR1 and CR2 datasets with the maximum likelihood (ML) reconstructed in IQ-TREE [33] using a model finder with the options TEST and –AICc, a tree search with 1000 bootstrap replicates, and Bayesian inference (BI) with MrBayes version 3.2.6 [34]. The GenBank database of four varanids is shown in Table 1 as of April 2021. The best-fit model of DNA substitution was determined for each CR using Kakusan4 [35]. The Markov chain Monte Carlo process was used to simultaneously run four chains for one million generations. After stabilization of the log-likelihood value, a sampling procedure was performed every 100 generations to obtain 10,000 trees, from which a majority-rule consensus tree with average branch lengths was generated. All sample points were discarded before attaining convergence as burn-in, and the Bayesian posterior probability in the sampled tree population was calculated as a percentage. The genetic distances of *p*-distance between CR sequences were calculated using the MEGAX program [22].

**Table 1:** Species used with accession numbers.

Species	GenBank Accession Number	CRs		Reference
		CR1	CR2	
*Varanus salvator macromaculatus*	LC326253-LC326324	CR1	-	This study
*Varanus salvator macromaculatus*	LC326325-LC326396	-	CR2	Wongtienchai et al. [5]
*Varanus salvator*	EU747731	CR1	CR2	Castoe et al. [36]
*Varanus salvator macromaculatus*	AB980995	CR1	CR2	Chaiprasertsri et al. [37]
*Varanus salvator komaini*	AB980996	CR1	CR2	Chaiprasertsri et al. [37]
*Varanus exanthematicus*	AB738957	CR1	CR2	-
*Varanus komodoensis*	AB080276	CR1	CR2	Kumazawa and Endo [11]
*Varanus niloticus*	AB185327	CR1	CR2	Kumazawa [13]

### 2.6. Recombination Testing

Discordant evolutionary signals were detected when the phylogenetic trees were separately reconstructed from different regions. These conflicting signals are due to the recombination of duplicate CRs. To further analyze these signals, the following recombination tests were conducted for both the CRs: (1) Recombination Detection Program (RDP) [38], (2) Geneconv [39], (3) Maxchi [40], and (4) Chimaera [41]. These analyses were performed using the Recombination Detection Program, RDP5 [42], with previously described parameters [43]. All analyses were performed for all individuals to check for recombination occurrence in both CRs of water monitors.

## 3. Results

### 3.1. Positional Annotation in the Control Regions of V. salvator macromaculatus

**Figure 1:**
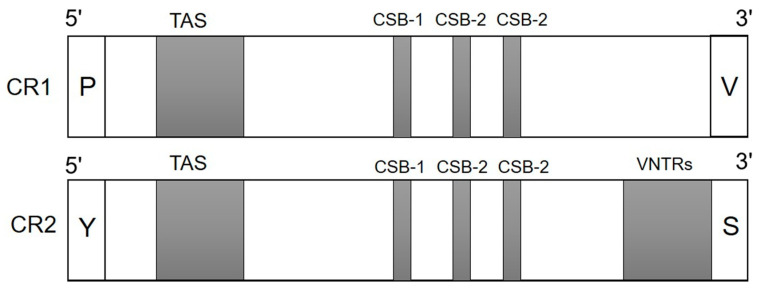
Structures of duplicate control regions (CRs) in all individuals of *Varanus salvator macromaculatus* (Deraniyagala 1944 [16]) in this study. Two functional regions, TAS and CSB, were detected in both the CRs of all individuals. The core sequences of these regions were found to be identical in both CR1 and CR2. Variable numbers of tandem repeats were detected only in CR2.

Three conserved functional sections, including the TAS, CD, and CSB domains, were analyzed in both CR1 and CR2 of all the 72 individuals; however, no CD was observed in either CR. The TAS domain contained 78 bp for CR1 and 80 bp for CR2 in water monitor lizards. The conserved nucleotide sequence of TAS between CR1 and CR2 was 5′-TAGTT-3′. The CSB domain contained three conserved blocks, namely CSB-1 (5′-TTAATGGTCDCNGGRHAT -3′), CSB-2 (5′-DHWDBYMYNYHHDCYYYC -3′), and CSB-3 (5′-GCYHWDYRKTYAHMMAA-3′) for CR1, and CSB-1 (5′-TTCATYWYHAWWWWTTBDN-3′), CSB-2 (5′-WWWWYCMYYWWHYYYY -3′), and CSB-3 (5′-GCYHWWYRKTYAHMAA -3′) for CR2. VNTRs were identified in CR1 (TCGCGCCACCTCCAGGATT), with two copies for one individual only, and CR2, (TTTTTTAAAAAAATTTTTTAT), (AAAAAAATTTTTTTA), (TTAAAAAAATTTTTT), and (AAAAAAATTTTTTATTTTTTTAA), ranging from to 2 to 4 copies in all individuals (Figure 1).

### 3.2. Sequence Variation in the CRs of V. salvator macromaculatus

**Figure 2:**
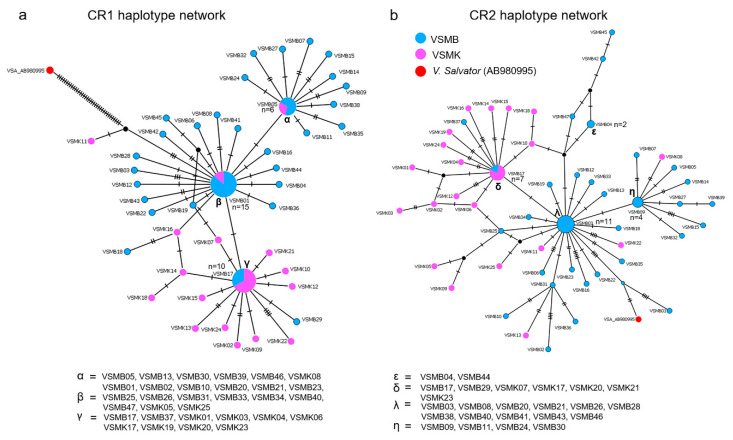
Haplotype network based on mitochondrial control region (mtCR) region sequence data of water monitors from Bang Kachao Peninsula (VSMB) and Varanus Farm Kamphaeng Saen (VSMK) populations, constructed using statistical parsimony with the TCS network. The numbers of individuals possessing haplotypes are indicated by different colors inside the circles. Missing haplotypes are indicated by black circles. (**a**) mtCR1 haplotype network (**b**) mtCR2 haplotype network.

The alignment lengths of CR1 and CR2 sequences were 663 and 867 bp, respectively. The number of haplotypes in CR1 was 44, and the number of haplotypes in CR2 was 52. The overall haplotype and nucleotide diversities were 0.935 ± 0.019 and 0.004 ± 0.001 for CR1 and 0.968 ± 0.013 and 0.004 ± 0.001 for CR2. Results of the *t*-test showed that the means of *h* and π significantly differed between CRs (t = 3.974, df = 141.77, *p*-value < 0.01 for *h* value and t = 6.8043, df = 127.83, *p*-value < 0.01 for π value). Meanwhile, results of the *t*-test showed that the means of *h* and π significantly differed between CSB (t = 32.678, df = 116.3, *p*-value < 0.01 for *h* value and t = 18.607, df = 126, *p*-value < 0.01 for π value) and TAS (t = −29.507, df = 125.8, *p*-value < 0.01 for *h* value and t = −9.779, df = 135.6, *p*-value < 0.01 for π value). Complex haplotype networks of both CR1 and CR2 were constructed from a large number of polymorphic sites and haplotypes, showing a striking star-shaped topology (Figure 2). The average sequence divergences between CR1 and CR2 (the paralogous CRs) of the same species (*p*-distance) were 0.39% ± 0.11% and 0.37% ± 0.10%, respectively, whereas those of orthologous CRs in different species were 10.83% ± 0.58% for CR1 and 17.08% ± 0.45% for CR2 (Table 2).

**Figure 3:**
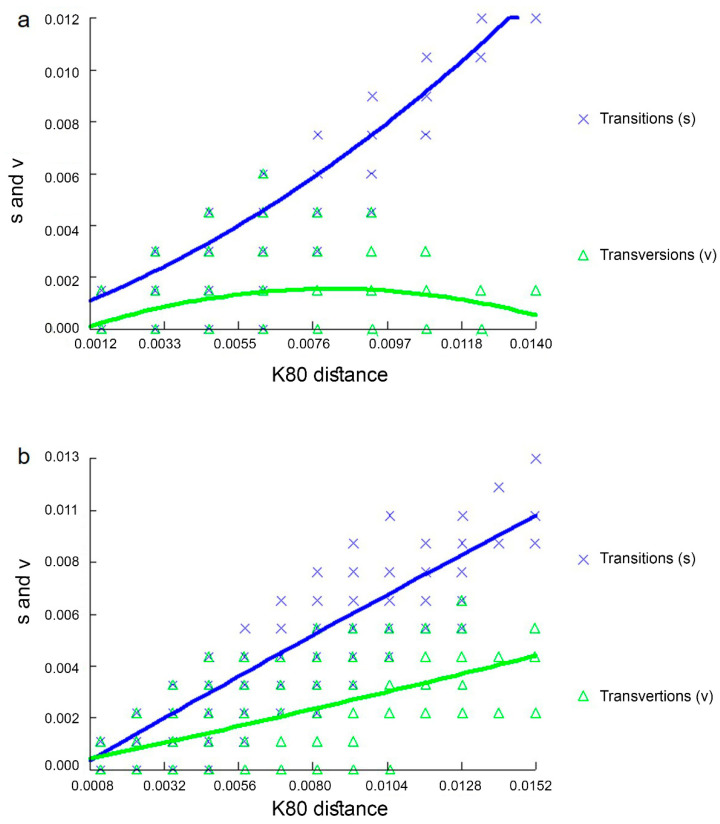
DAMBE7 substitution saturation plots for (**a**) CR1 and (**b**) CR2. Numbers of transitions (s) and transversions (v) are plotted against the K80 distance; lines indicate mean values (thick lines) and standard deviations (fine lines) of s and v.

Substitution saturation was estimated for both CR1 and CR2 datasets. No saturation was detected in CR2, as reflected by the linear correlation of the number of transitions and transversions plotted against sequence divergence (Figure 3) as well as from a significantly lower value of I_ss_ as compared to I_ss.c_ (Table 3). By contrast, in CR1, the number of transitions was higher than that of transversions, and substitution saturation occurred when the frequency of transitions exceeded the frequency of transversions (Figure 3).

### 3.3. Phylogenetic Relationship Based on the Control Regions of V. salvator macromaculatus

Phylogenetic analyses based on CR1 and CR2 were supported with high posterior probabilities and bootstrap values. Although the phylogenetic trees shared similar topologies, general differences existed (Supplementary Figures S1–S4). When combining both CR sequence datasets, paralogous CRs of an individual in the same dataset did not group together, while orthologous CRs of different individuals always clustered. When reconstructed separately using the separated regions (Supplementary Figures S5–S10), phylogenetic trees of TAS and CSB sequences shared the same topologies, indicating that orthologous copies from different individuals always clustered together rather than with paralogous copies from the same individuals. However, CR1 and CR2 from the same species are always clustered together (Supplementary Figures S1–S4).

### 3.4. Recombination Events in CRs

**Figure 4:**
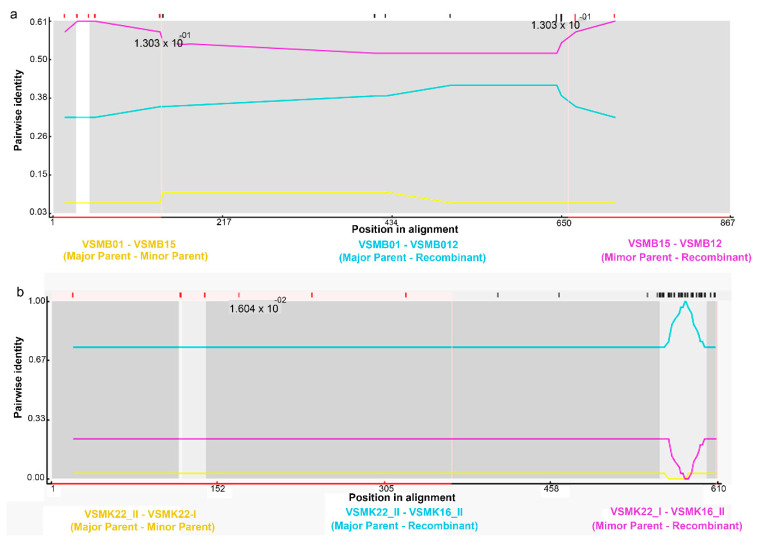
Assessment of recombination in duplicate CRs of all individuals: (**a**) CR2; (**b**) CRs detected using RDP software.

To explain the presence of discordant signals between phylogenetic trees derived from CR1 and CR2 constructs, multiple recombination points were investigated using RDP software (Figure 4). Non-significant recombination events in CRs were observed at positions 138 bp and 672 bp in CR2 (MaxChi, *p* = 0.137; RDP, Geneconv, and Chimaera, no evidence of recombination was found) whereas no events were found in CR1. However, when we combined CR1 and CR2 of all individuals, a recombination event was observed at position 555 (Geneconv, *p* < 0.05) while no evidence of recombination was found in RDP, MaxChi, and Chimaera.

## 4. Discussion

Vertebrate mitogenome sequences are important systems that are predominantly utilized for molecular evolutionary studies, phylogenetics, and systematic taxonomy [3,5,7,44,45,46,47,48,49,50]. A special phenomenon has been observed in mitogenomes involving several vertebrates with duplicate CRs [9]. Comparisons of the four mitogenomes of varanids have revealed that all species possess duplicate CRs that tally with the process of mitogenomic rearrangement, and they can be reshuffled by investigating the use of PCR-based DNA marker analysis across 11 varanids [15]. The CR structures in vertebrate mitogenomes predominantly contain conserved sequences known as TASs and CSBs, which were observed in both CR1 and CR2. These conserved sequences are known to play important roles in the replication and expression of mitogenomic genes [6,51]. However, no CD was observed for either CR1 or CR2. This result suggests the plasticity of mitogenomes with the CD motif across vertebrates.

Entire sequences of CRs within species were highly similar, and the paralogs of CR1 and CR2 from each species showed a closer resemblance than those of their orthologs from other lineages. This suggests that CRs concertedly evolved in each species [9]. Duplicated CRs might remain conserved during reproduction and thus maintained in the mitogenome during speciation of varanids [6]. Alternatively, duplicate CRs probably play different roles in the replication of mitogenomes under evolutionary selective pressure and may have evolved independently within a particular species [52]. We observed this appearance in sequence divergences between CR1 and CR2 in 72 *V. salvator macromaculatus* individuals from the two populations [5]. The observed values were 5.91% and 17.4% between the two CRs. Homologs of CRs from different individuals were genetically more similar than paralogous CRs from the same individual within a species (Supplementary Figure S3), thereby agreeing with phylogenetic analyses, although both populations studied exhibited a high degree of population-level genetic diversity [5]. This suggests the independent evolution of evolutionary patterns for duplicate CRs within *V. salvator macromaculatus*. The CRs would have evolved independently within each varanid species after the divergence of species as a result of different mutations between CR1 and CR2 during evolution.

However, with evolutionary variations in characteristics, we found evidence of substantial variation in the inferred usage of CRs in *V. salvator macromaculatus*. Saturation analyses also showed that plots of transitions and transversions related linearly, with sequence divergence indicating no saturation in the CR2 data set [53]. The non-linear pattern in the CR1 data set suggested substitution saturation (Figure 3); therefore, CR2 may be a more informative phylogenetic marker at the population level in varanids. Different mitochondrial genome coding regions with diverse mutation rates and evolutionary trajectories are required to further elucidate the varanid phylogenetic lineage. Further analysis on their differential expression should be performed across varanid species to clearly understand the role of duplicate CRs. Although phylogenetic trees of CR1 and CR2 shared generally similar topologies, only minor differences existed in the placement of specimens (Supplementary Figures S1–S10). Additionally, ML and BI trees were constructed based on the TAS and CSB datasets derived from all individuals with three other variants. Phylogenetic trees of TAS and CSB sequences were largely congruent with each other, and homologs within an individual always formed closer clusters than their paralogs from other individuals. The phylogenetic analysis detected certain discordant signals among different CRs, suggesting that recombination might have reshaped the evolution of duplicate CRs in the mitogenomes of water monitors. The results of this study provide several recombination points for CR2. The breakpoints of recombination tend to occur at 138 bp and 672 bp in the TAS motif. It is essential to further investigate the recombination sites of CRs to better understand the evolutionary conflict and accurately detect the phylogenetic patterns [6,54,55]. This can be achieved by analyzing heterologous sequences that contribute to mitogenomic recombination [6]. In the mtDNA of water monitors, heterogeneous regions (A and T arrays) were detected in the VNTR motif [56]. There are four types of compound CT arrays. Different studies have identified the mitogenomic recombination sites associated with heterologous in various vertebrates [6,56,57]. Specifically, VNTRs in CR2 of different species or individuals were heterologous with respect to their sequence size and motifs. This suggests that VNTRs have recombination roles. Further analyses with more varanid species are required to investigate more basic questions such as how mitochondrial genomes with duplicate CRs evolved and how the nucleotide sequences of duplicate CRs remained identical or highly similar over evolution under the concept of concerted and independent evolution.

## 5. Conclusions

This paper reports foundational knowledge on the dynamics of duplicated CRs in varanids mitogenomes. Our data suggest that these sequences might follow independent evolution within the same species. CRs seem to have acquired concerted evolution across different species. This hypothesis provides a baseline to study mitogenomic evolutionary events such as recombination, gene rearrangement, and concerted evolution between duplicates. A thorough understanding of nucleotide substitution in varanid CRs is important for advances in evolutionary model construction, with accurate evolutionary inferences to provide basic insights into the biology of mitogenomes.

## Figures and Tables

**Table 2:** Percentage of D-loop sequence diversity for Asian water monitor (*Varanus salvator macromaculatus*; Deraniyagala 1944).

Region	Within Group	Between Group
	*p*-Distance	*p*-Distance
CR1	0.39 ± 0.11	-
CR2	0.37 ± 0.10	-
CSB_CR1	0.40 ± 0.18	-
CSB_CR2	0.15 ± 0.09	-
TAS_CR1	011 ± 0.11	-
TAS_CR2	0.21 ± 0.08	-
CR1 *	10.83 ± 0.58	25.11 ± 5.50
CR2 *	17.08 ± 0.45	38.88 ± 1.54
CSB_CR1 *	11.23 ± 1.96	34.70 ± 6.40
CSB_CR2 *	14.33 ± 1.88	32.49 ± 6.00
TAS_CR1 *	13.78 ± 0.17	41.10 ± 5.50
TAS_CR2 *	17.08 ± 0.45	38.88 ± 1.55

* Comparison between CRs of *Varanus salvator macromaculatus* with CRs of *V. salvator*, *V. salvator macromaculatus*, *V. salvator komaini*, *V. exanthematicus*, *V. komodoensis*, and *V. niloticus*.

**Table 3:** Substitution saturation analysis of CRs based on the index of substitution saturation as implemented in DAMBE7 [32].

Region	Number of OUT ^a^	I_ss_^b^	I_ss.cSym_^c^	Df ^d^	*p*-Value ^e^	I_ss.cAsym_^f^	df	*p*-Value
CR1	4	0.028	0.805	662	<0.00001	0.774	662	0.0000
	8	0.033	0.766	662	<0.00001	0.656	662	0.0000
	16	0.039	0.745	662	<0.00001	0.535	662	0.0000
	32	0.046	0.719	662	<0.00001	0.393	662	0.0000
CR2	4	0.007	0.815	866	<0.00001	0.784	866	0.0000
	8	0.008	0.780	866	<0.00001	0.673	866	0.0000
	16	0.008	0.763	866	<0.00001	0.560	866	0.0000
	32	0.009	0.738	866	<0.00001	0.424	866	0.0000

^a^ number of sequences used in random resampling; OTP: operational taxonomic unit, ^b^ index of substitution saturation, ^c^ critical value for a symmetrical tree topology, ^d^ degrees of freedom, ^e^ probability that I_ss_ is significantly different from the critical value (I_ss.cSym_/I_ss.cAsym_), ^f^ critical value for an asymmetrical tree topology.

## Data Availability

DNA sequences: GenBank accessions LC315243–LC315386. Microsatellite data submitted: Dryad (https://doi.org/10.5061/dryad.v6wwpzgt4).

## Supplementary Materials

The following are available online at https://www.mdpi.com/article/10.3390/ani12020148/s1: Figure S1: Phylogenetic relationship between all individual water monitors (*Varanus salvator macromaculatus*; Deraniyagala 1944) and one GenBank accession: AB167711 constructed with the help of Bayesian inference (BI) analysis using CR1 sequence. Support values at each node are bootstrap values of Bayesian posterior probability. Figure S2: Phylogenetic relationship between all individual water monitors (*Varanus salvator macromaculatus*; Deraniyagala 1944) and one GenBank accession: AB167711 constructed with the help of Bayesian inference (BI) analysis using CR2 sequence. Support values at each node are bootstrap values of Bayesian posterior probability. Figure S3: Phylogenetic relationship between all individual water monitors (*Varanus salvator macromaculatus*; Deraniyagala 1944) and one GenBank accession: AB167711 constructed with the help of Bayesian inference (BI) analysis using CRs sequence. Support values at each node are bootstrap values of Bayesian posterior probability. Figure S4: Phylogenetic relationship between all individual water monitors (*Varanus salvator macromaculatus*; Deraniyagala 1944) and one GenBank accession: AB167711 constructed with the help of maximum likelihood (ML) analysis using of CRs sequence. Support values at each node are bootstrap values of maximum likelihood posterior probability. Figure S5: Phylogenetic relationship between all individual water monitors (*Varanus salvator macromaculatus*; Deraniyagala 1944) and one GenBank accession: AP018114 constructed with the help of Bayesian inference (BI) analysis using CSB of CR1 sequence. Support values at each node are bootstrap values of Bayesian posterior probability. Figure S6: CSB CR2: Phylogenetic relationship between all individual water monitors (*Varanus salvator macromaculatus*; Deraniyagala 1944) and one GenBank accession: AP018114 constructed with the help of Bayesian inference (BI) analysis using CSB of CR2 sequence. Support values at each node are bootstrap values of Bayesian posterior probability. Figure S7: CSB CRs: Phylogenetic relationship between all individual water monitors (*Varanus salvator macromaculatus*; Deraniyagala 1944) and one GenBank accession: AP018114 constructed with the help of Bayesian inference (BI) analysis using CSB of CRs sequence. Support values at each node are bootstrap values of Bayesian posterior probability. Figure S8: TAS CR1: Phylogenetic relationship between all individual water monitors (*Varanus salvator macromaculatus*; Deraniyagala 1944) and one GenBank accession: AP018114 constructed with the help of Bayesian inference (BI) analysis using TAS of CR1 sequence. Support values at each node are bootstrap values of Bayesian posterior probability. Figure S9: TAS CR2: Phylogenetic relationship between all individual water monitors (*Varanus salvator macromaculatus*; Deraniyagala 1944) and one GenBank accession: AP018114 constructed with the help of Bayesian inference (BI) analysis using TAS of CR2 sequence. Support values at each node are bootstrap values of Bayesian posterior probability. Figure S10: TAS CRs: Phylogenetic relationship between all individual water monitors (*Varanus salvator macromaculatus*; Deraniyagala 1944) and one GenBank accession: AP018114 constructed with the help of Bayesian inference (BI) analysis using TAS of CRs sequence. Support values at each node are bootstrap values of Bayesian posterior probability. Table S1: Summary of water monitor lizard (*Varanus salvator macromaculatus*) specimens.
