# Supplementary material for: Concerted and Independent Evolution of Control Regions 1 and 2 of Water Monitor Lizards (Varanus salvator macromaculatus) and Different Phylogenetic Informative Markers
Source: Animals (Basel). 2022 Jan 8;12(2):148. doi: 10.3390/ani12020148 (PMC8772547; doi:10.3390/ani12020148)
Supplement: Supplementary file 1 [file animals-12-00148-s001.zip › Supplementary Table S1.pdf]

Table S1 Summary of water monitor lizard (*Varanus salvator macromaculatus*) specimens. (Wongtienchai et al. 2021)

Locality	Geographic coordinates	Number of water monitor lizard samples	Code	CR1 GenBank accession number	CR2 GenBank accession number
Bang Kachao Peninsula	13°41'38"N, 100°33'39"E	47	VSMB01-VSMB47	LC326253 – LC326299	LC326325– LC326371
Varanus Farm Kamphaeng Saen	13°59′2″N, 99°59′38″E	25	VSMK01-VSMK25	LC326300 – LC326324	LC326372 – LC326396

Wongtienchai, P.; Lapbenjakul, S.; Jangtarwan, K.; Areesirisuk, P.; Mahaprom, R.; Subpayakom, N.; Singchat, S.; Sillapaprayoon, S.; Muangmai, N.; Songchan, R.; et al. Genetic management of a water monitor lizard (*Varanus salvator macromaculatus*) population at Bang Kachao Peninsula as a consequence of urbanization with Varanus Farm Kamphaeng Saen as the first captive research establishment. *J. Zoolog. Syst. Evol. Res.* **2021**, *59*, 484–497.
