# Supplementary figures and images for: Concerted and Independent Evolution of Control Regions 1 and 2 of Water Monitor Lizards (Varanus salvator macromaculatus) and Different Phylogenetic Informative Markers
Source: Animals (Basel). 2022 Jan 8;12(2):148. doi: 10.3390/ani12020148 (PMC8772547; doi:10.3390/ani12020148)

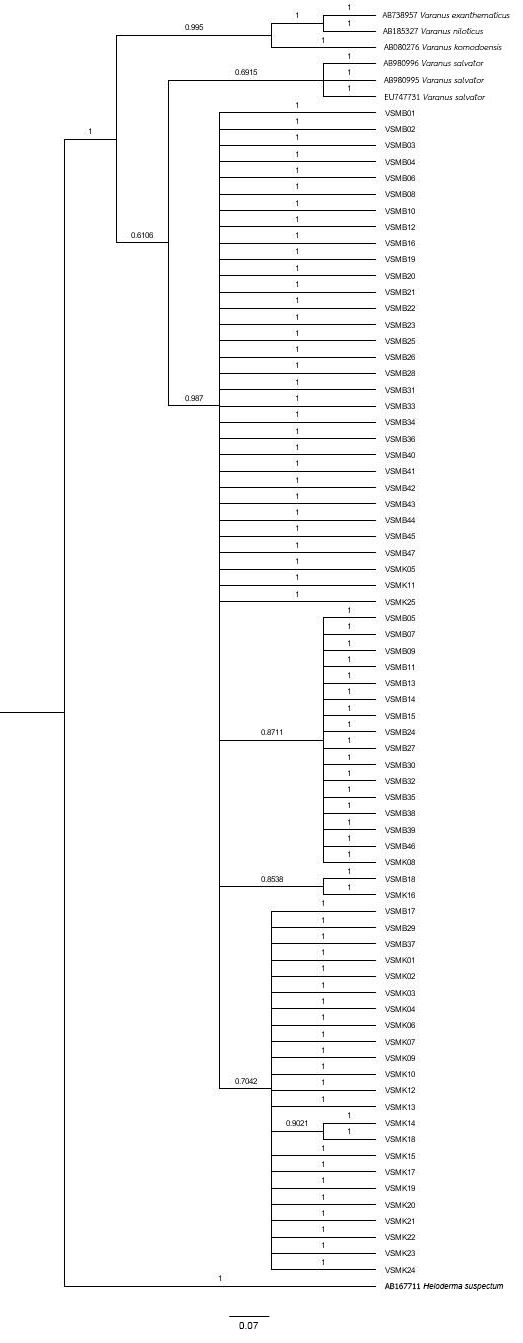

Supplement: Supplementary file 1 [file animals-12-00148-s001.zip › Figure S1.tif]

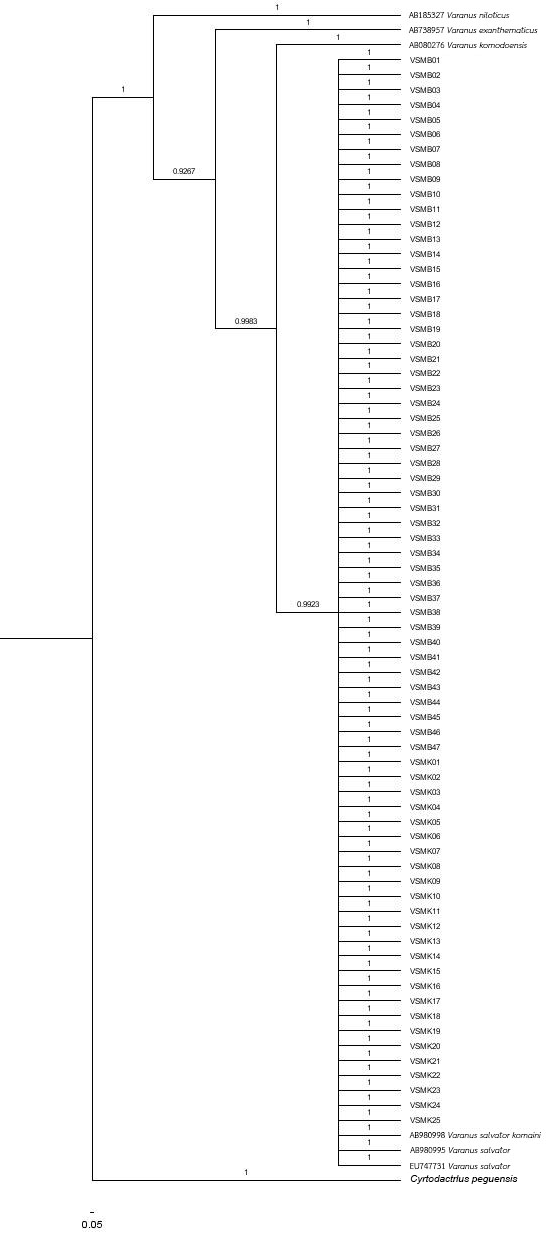

Supplement: Supplementary file 1 [file animals-12-00148-s001.zip › Figure S10.tif]

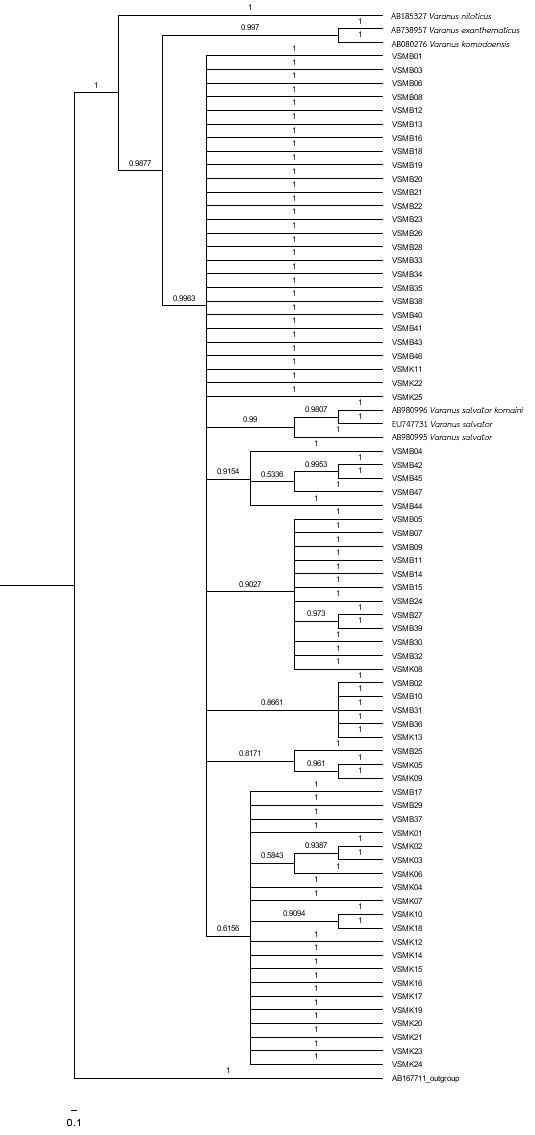

Supplement: Supplementary file 1 [file animals-12-00148-s001.zip › Figure S3.tif]

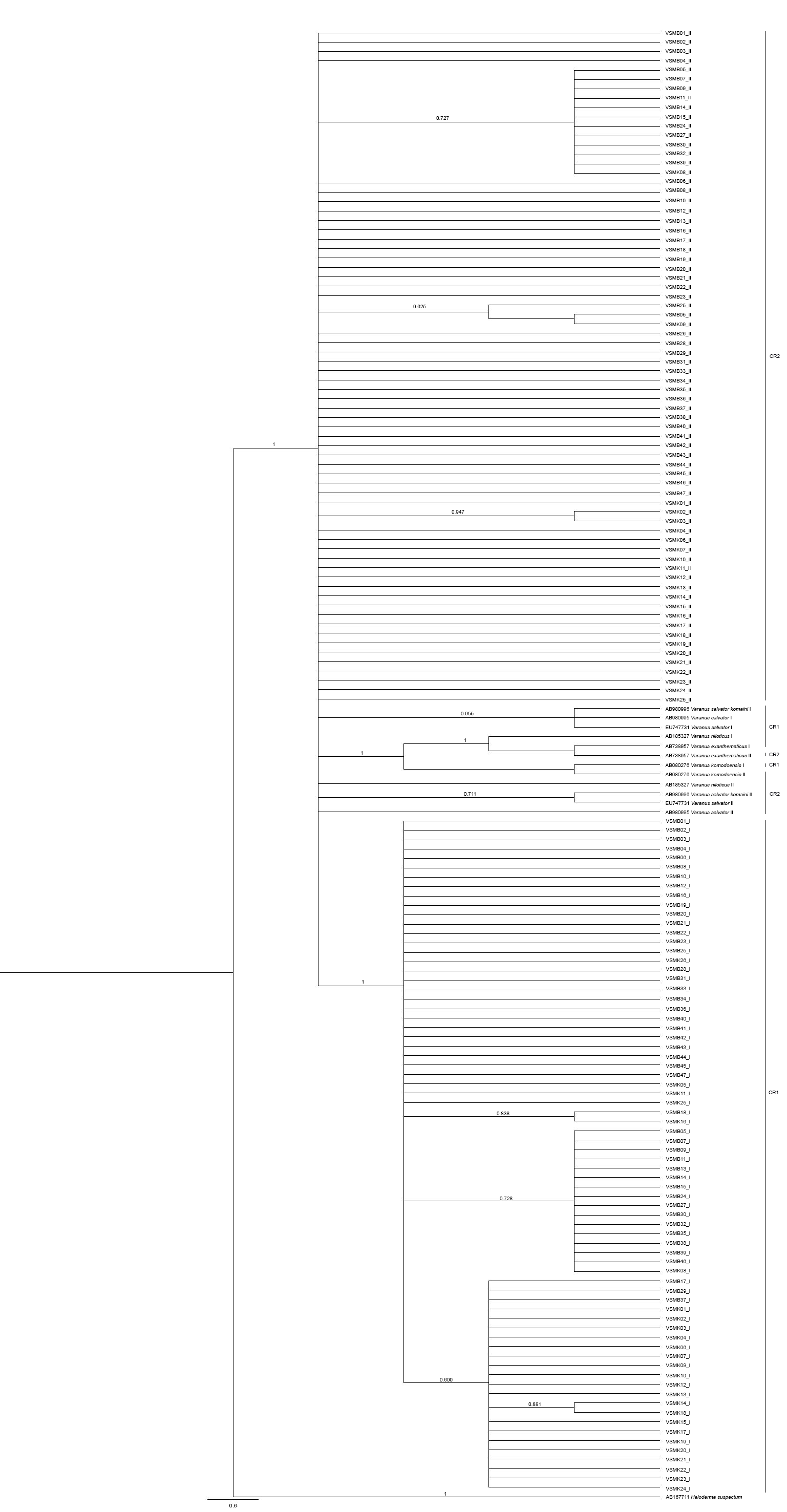

Supplement: Supplementary file 1 [file animals-12-00148-s001.zip › Figure S4.tif]

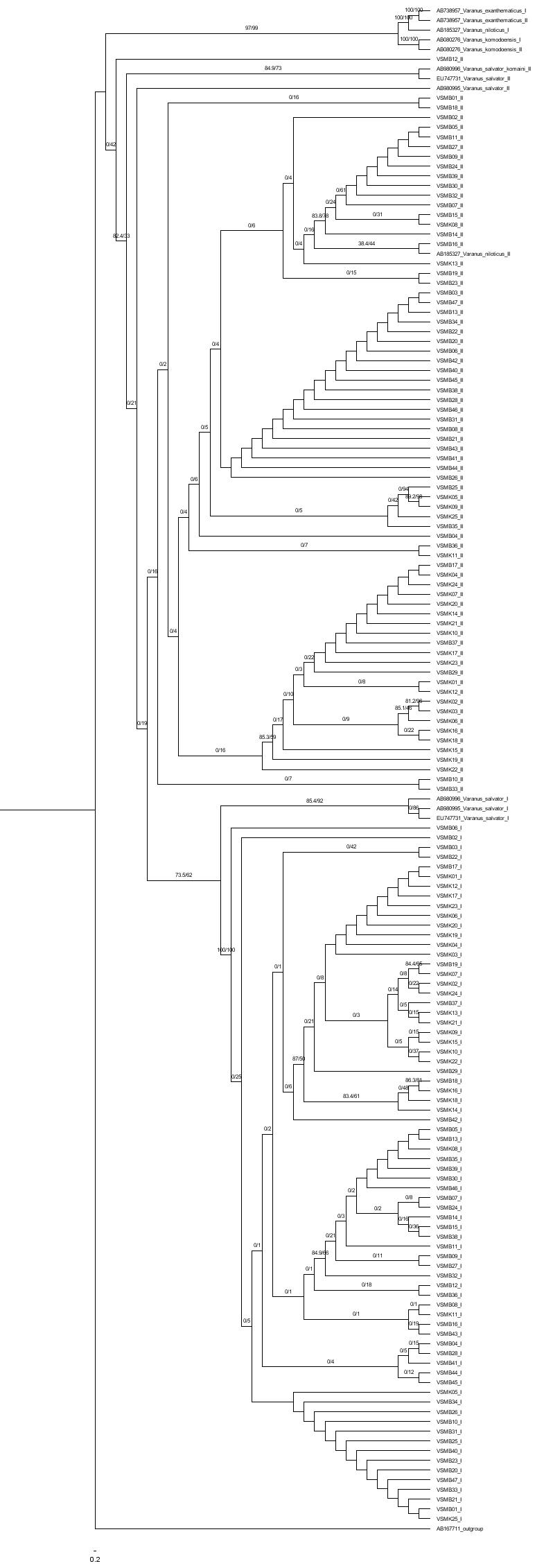

Supplement: Supplementary file 1 [file animals-12-00148-s001.zip › Figure S5.tif]

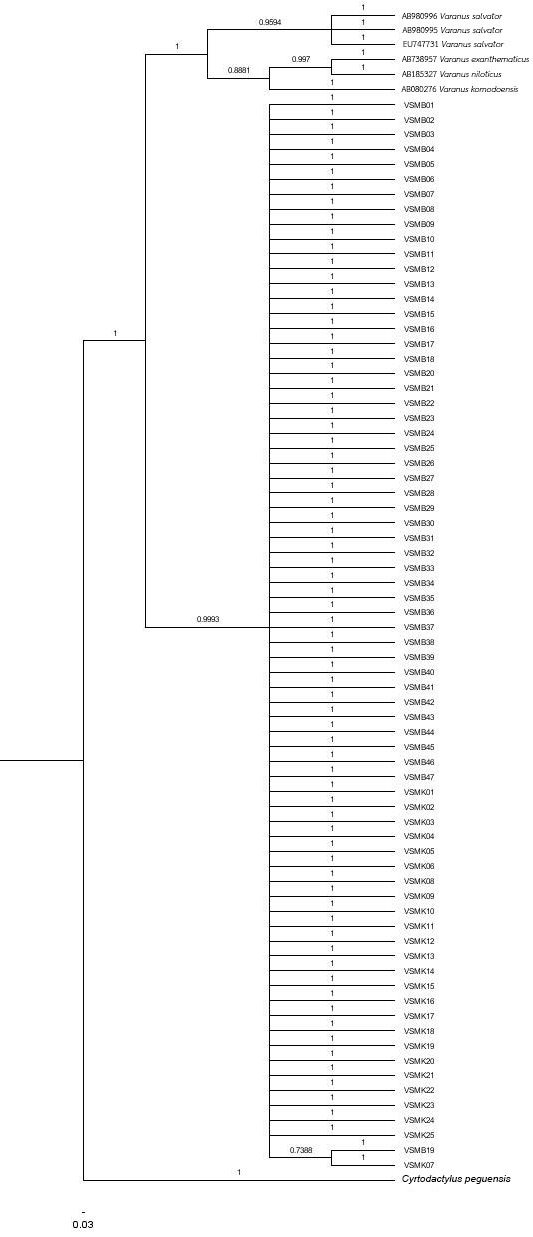

Supplement: Supplementary file 1 [file animals-12-00148-s001.zip › Figure S6.tif]

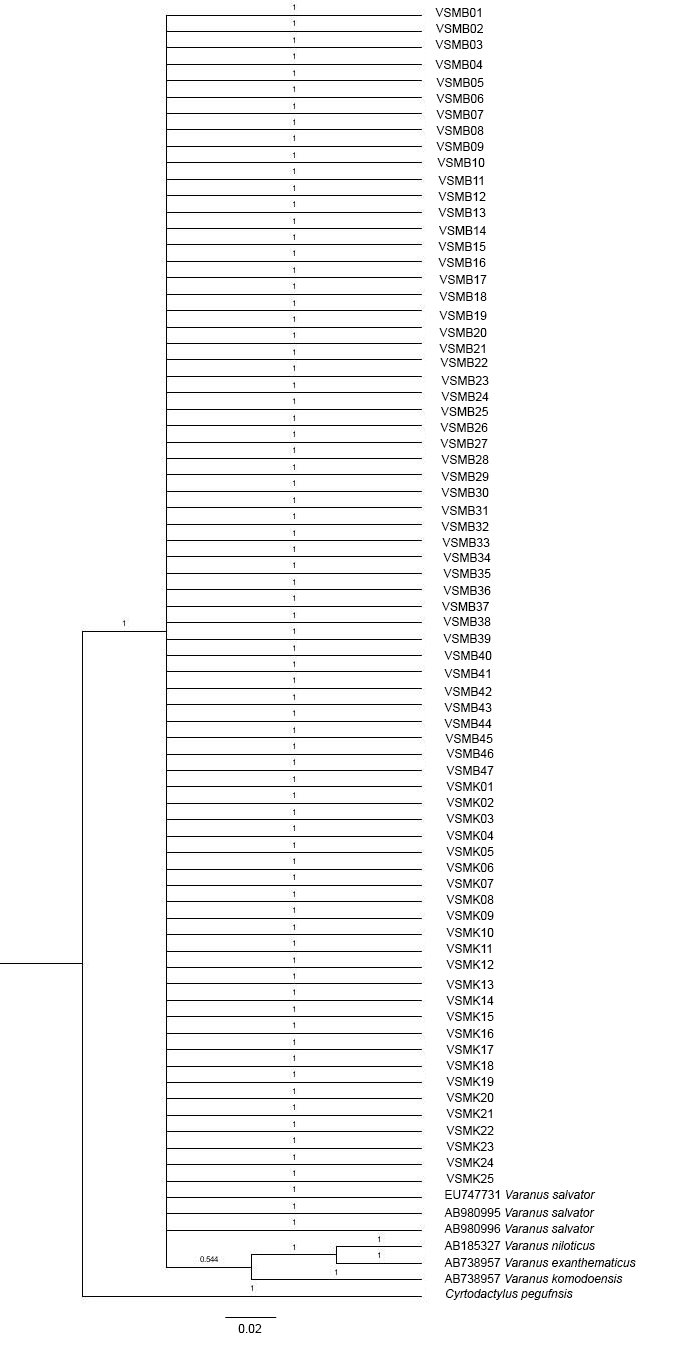

Supplement: Supplementary file 1 [file animals-12-00148-s001.zip › Figure S7.tif]

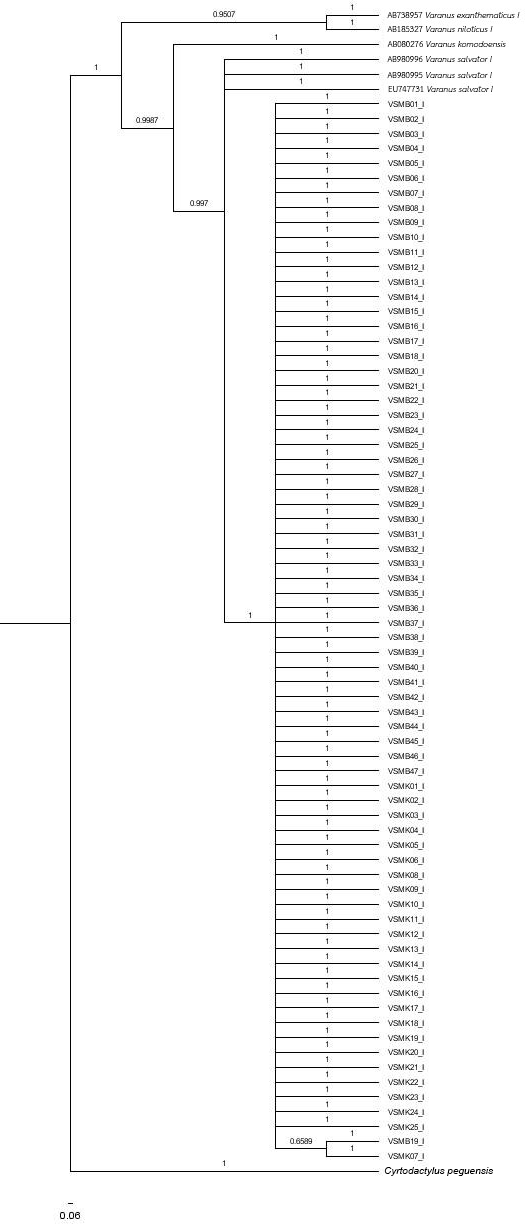

Supplement: Supplementary file 1 [file animals-12-00148-s001.zip › Figure S9.tif]
